# Dry-Matter Loss and Changes in the Chemical Composition of Spruce Wood after Long-Term Storing in the Form of Roundwood

**DOI:** 10.3390/polym14163400

**Published:** 2022-08-19

**Authors:** Richard Hrčka, Viera Kučerová, Vladimír Hönig

**Affiliations:** 1 Department of Wood Sciences, Faculty of Wood Sciences and Technology, Technical University in Zvolen, T.G. Masaryka 24, 96001 Zvolen, Slovakia; 2 Department of Chemistry and Chemical Technologies, Faculty of Wood Sciences and Technology, Technical University in Zvolen, T.G. Masaryka 24, 96001 Zvolen, Slovakia; 3 Department of Chemistry, Faculty of Agrobiology, Food and Natural Resources, Czech University of Life Sciences Prague, Kamýcká 129, 169 21 Prague, Czech Republic

**Keywords:** spruce wood, roundwood storage, dry-matter loss, holocellulose, cellulose, FTIR

## Abstract

Wood stock in a warehouse is a necessary precondition for reliable manufacturing. However, wood can degrade and lose the matter during storage. “Dry-matter loss (DML)” is used to quantify the degradation following the changes in mass of a wood substance. The proposed calculation of DML is based on using parallel figures. The calculated loss of spruce wood substance harvested in winter during a six-month period was 4.5%. The estimated annual loss of wood substance was 5.7%. The loss was caused by a factor with a gradually eliminated effect. The changes in the chemical composition of wood substance were not proportional to the original amount of the isolated chemical substances. Hemicelluloses and lignin suffered from the loss faster than there was a change in the DML of spruce wood. Hemicelluloses were the most unstable isolated compound, with an increased rate of change during the first four months. The number of extractives significantly decreased during two months of storage. However, there was an increase in the number of extractives after six months of storage. The loss of cellulose was similar to the DML of spruce wood during the whole time of storage. The FTIR analysis confirmed a decrease in the total crystalline index (TCI) and lateral order index (LOI) of cellulose due to the storage of roundwood.

## 1. Introduction

Long-term storage of round wood is inevitable in the production of cellulose due to the need for the uninterrupted supply production process with wood and the market-driven decisions of industrial buyers. However, wood degradation and loss of the wood substance during storage can be considered a problem as it is also mentioned in the works summarizing the facts about “Dry-matter loss (DML)” [1,2]. Internal and external factors are the main aspects affecting the wood substance loss. External biotic factors are wood-destroying and wood-staining fungi, bacteria, insects, etc. [3]. The common wood-decaying fungus, *Trametes versicolor*, can cause a loss of 46% in the mass of spruce wood in six months [4]. The coefficient of variation of the loss of wood substance in the mentioned research was 12%. However, the research was conducted under ideal conditions, with just one species, at optimum temperature and humidity. Therefore, the mass losses of wood substance are great. The extent to which the wood decomposes depends on how long it is stored outside [5]. Biological degradation results in the loss of wood substance and reduction of wood quality, i.e., a decrease in strength properties of fibres, change in colour causing difficulties in further processes and economic loss [5,6]. It occurs when the amount of water in moist wood ranges from 25% to 55% [5]. The mass loss of roundwood in a heap of wood stock is approximately 2–3% during the first year of storage but less than 0.1% when stored in water [5]. Abiotic external factors are catalysts of chemical reactions. Temperature, relative air humidity, and precipitation are climatic conditions presented in relation to DML [1,7]. Biotic internal factors affecting the DML are linked to living wood cells after felling and shrinkage of cytoplasm in the wood cells. We think the cambium cell (cambial initials), tracheid of the last growth ring, and parenchymatic cells are alive after felling. Live parenchymatic cells are in sapwood because of its higher moisture content (significantly higher amount of bound water) and in roundwood as cells of wood parenchyma [8]. Chovanec et al. [8] mention that there is an inconsiderable amount of wood parenchyma in spruce wood. In spruce wood, there is 90% of tracheids. Then, in the total volume of spruce wood, there is 10% of the parenchyma of pith rays [9]. The ratio of spruce sapwood was investigated by Millers [10]. Following his results, the total volume of spruce wood contains 51 to 70% sapwood. The loss of DML caused by external abiotic factors is due to the diffusion of volatile substances evaporating from wood. He et al. describe the evaporating of CO_2_, CO, CH_4_, and other volatile organic substances such as methane [11,12]. The authors stated that the reason for DML and the release of volatile substances from wood in the form of gases occurs during the activity of abiotic and biotic factors. Gigler et al. [13], Jensen et al. [14], Salin and Gjerdrum [15], and Erber et al. [16] present the loss of the total wood mass together with the loss of wood moisture. Routa et al. [17] mention the lower value of DML in the case of lower moisture (area of bound water), even though the moisture content increases later during storage (to the area of free water).

Wood consists of wood substance, water, and air [18]. The mass of the air inside wood is negligible as wood is weighted on air. The mass of wood depends mainly on the water content in the wood’s volume and the wood substance’s mass. Those are properties associated with the state of wood. Two hundred unique properties of wood were detected by Sydor and Wieloch (2009) [19]. The moisture content measurement method needs only two of them: mass of water and mass of wood substance. Wood substance mass is determined with the oven-dried method as mass of oven-dried wood.

According to Assarsson et al. [20], DML equals 1.1% after a month of storage of the spruce roundwood in the summer. Routa et al. [21] mention the range of DML from 0.07% to 1.52% after one-month storage of roundwood. Nilsson et al. [7] determined DML over six months—from January to June with a total value of 5.6% (monthly 0.93%). According to Sixta [5], the value of DML for roundwood is 2–3%, but less than 0.1% when stored in or underwater.

This study aims to determine the dry-matter loss (DML) of round spruce wood stored for a one year in a warehouse. The wood contains various substances; therefore, the changes in the chemical composition of wood after 2, 4, and 6 months of storage are also monitored in the paper. DML was estimated using a regression model after a year of storage.

## 2. Materials and Methods

It is supposed that the change in the mass of wood consists of two changes: the change in the wood substance mass and the change in the mass of water in the total volume of wood:

(1) $$\;dm=\frac{\partial m}{\partial m_{s}}dm_{s}+\frac{\partial m}{\partial m_{H2O}}dm_{H2O}$$

The change in the mass of the wood substance is the change in the mass of dry wood and it is defined by “dry-matter loss”, abbreviated *DML* [19]:

(2) $$DML=\left(1-\frac{m_{s2}}{m_{s1}}\right)100\%$$

where *m_s_*_1_, or *m_s_*_2_ are the masses of the wood substance before or after the action of the factor causing the loss of wood substance. (Note: Barontiny et al. [22] defined *m_s_*_1_, or *m_s_*_2_ as a mass of the wood substance before and after storage). The mass of the wood substance when the density varies in the dry state is calculated using the following formula:

(3) $$m_{s}=\overset{V_{0}}{\underset{0}{\int }}\rho_{0}dV_{0}$$

where *ρ*_0_ is the density in the absolutely dry state (oven-dried density) and *V*_0_ is the measured volume in the absolutely dry state. As in the initial state of wood, there is water together with the wood substance. The mass of absolutely dry wood cannot be determined without the information about the dry state. Therefore, the parallel figures must be used to determine *DML*. In the case of parallel figures, constant properties not dependent on coordinates at the beginning of an experiment are assumed. Parallel figures are small test samples with no visible defects, in regular shape with growth rings parallel to one side of the figure. The density in the dry state used to calculate the mass of the wood substance is a property of parallel figures necessary to determine *DML*:

(4) $$DML_{j}=1-\frac{2\sum_{i=1}^{N2j}\rho_{0i}\left({\left(\frac{R_{i+1}}{R_{max2}}\right)}^{2}-{\left(\frac{R_{i}}{R_{max2}}\right)}^{2}\right)}{\sum_{i=1}^{N1j}\rho_{0i}\left({\left(\frac{R_{i+1}}{R_{max1}}\right)}^{2}-{\left(\frac{R_{i}}{R_{max1}}\right)}^{2}\right)+\sum_{i=1}^{N2j}\rho_{0i}\left({\left(\frac{R_{i+1}}{R_{max2}}\right)}^{2}-{\left(\frac{R_{i}}{R_{max2}}\right)}^{2}\right)}$$

(5) $$DML=\overset{N}{\underset{j=1}{\sum }}DML_{j}$$

where *DML_j_* is the loss of the wood substance in the *j* part of roundwood. *N* is the number of roundwood, *ρ*_0*i*_ is the oven-dried density of the *i* figure with the distance of a radius *R_i_*, *R*_*max*1_, or *R*_*max*2_ (radius of roundwood from which parallel figures with the number of *N*_1*j*_ or *N*_2*j*_ were cut) from the pith.

**Figure 1:**
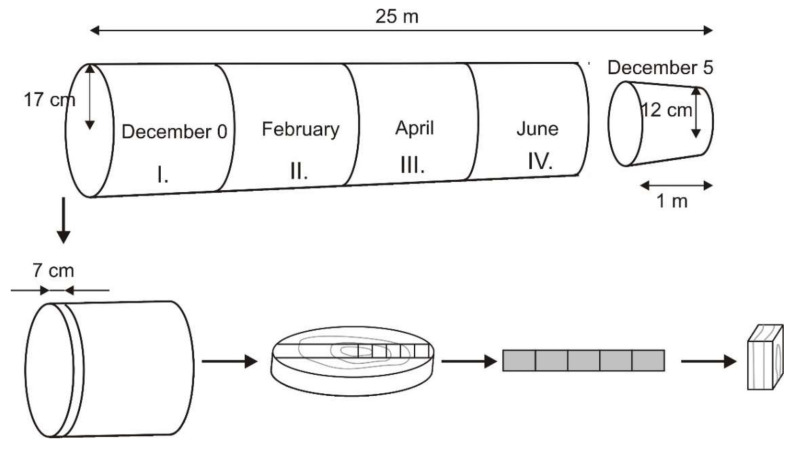
Scheme of test figure production.

Spruce wood (*Picea abies* L. Karst) was grown in Lukové (Slovakia). The spruce was felled in December at the height of 0.5 m from the earth’s surface and the sample December 0 was handled in the length of 1 m from the butt cut just after felling. The total length of the roundwood was 25 m. Subsequently, three pieces of roundwood with a length of 1 m were handled apart from the sample named as December 0. They were stored for 2, 4 and 6 months. The sample December 5 was cut 24 m from the butt cut. The estimated age of the spruce was 65 years. During storage, the temperature, relative humidity, and precipitation were measured. A disc with the thickness of 7 cm was cut from the samples. Rectangle-shaped test samples with dimensions of 20 × 20 × 30 mm (radial, tangential, and longitudinal anatomical directions) were cut from the disc (Figure 1). From the rest part of the disc, the sample in the shape of “V” was cut and subjected to chemical analyses.

Test figures were produced from the second, third, as well as fourth part of the roundwood. These three parts were debarked after 2, 4, and 6 months of storage before test figure production.

The moisture content was determined according to the standard STN EN 490103 [23] using the gravimetric method. Density at the given moisture content was measured according to the standard STN EN 490108 [24].

### 2.1. Chemical Composition of Spruce Wood

The wood samples were disintegrated to sawdust. The fraction of sawdust from 0.5 to 1.0 mm was used for the chemical analyses. Wood sawdust was extracted in the Soxhlet apparatus by a mixture of ethanol and toluene according to the ASTM D1107-96 [25]. The content of holocellulose was determined using the method by Wise et al. [26]. The content of cellulose was determined according to the method by Seifert [27]. Hemicellulose content was calculated as the difference between the content of holocellulose and cellulose. The content of lignin was determined according to ASTM D1106-96 [28]. All measurements were performed on three replicates per sample. Data are presented as percentages of oven-dry mass per unextracted wood.

### 2.2. Fourier-Transform Infrared Spectroscopy (FTIR)

Fourier-transform infrared spectroscopy (FTIR) absorbance spectra of holocellulose and cellulose were investigated within the 4000–650 cm^−1^ range (32 scans, 4 cm^−1^ resolutions, absorption mode). The experiment was performed using a Thermo Scientific Nicolet iS10 spectrometer (Thermo Fischer Scientific Instruments, Waltham, MA, USA) equipped with a diamond Smart iTR ATR sampling accessory. All analyses were performed in four replicates. The spectra were evaluated using the OMNIC 8.0 software (Thermo Fischer Scientific Instruments, Waltham, MA, USA).

The following parameters of cellulose were calculated: total crystalline index (TCI), lateral order index (LOI), as a ratio between the band intensities, respectively: 1370/2900 cm^−1^, 1424/898 cm^−1^.

## 3. Results and Discussion

**Figure 2:**
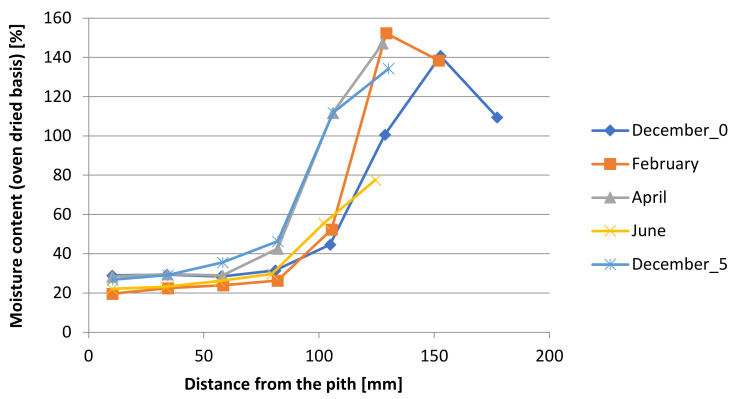
Moisture content in dependence on distance from the pith and during 6-month-long storage.

The change in the moisture content was investigated after different storage times of 0, 2, 4 and 6 months. Gradual water loss in wood is evident, especially at the beginning of summer (Figure 2).

**Figure 3:**
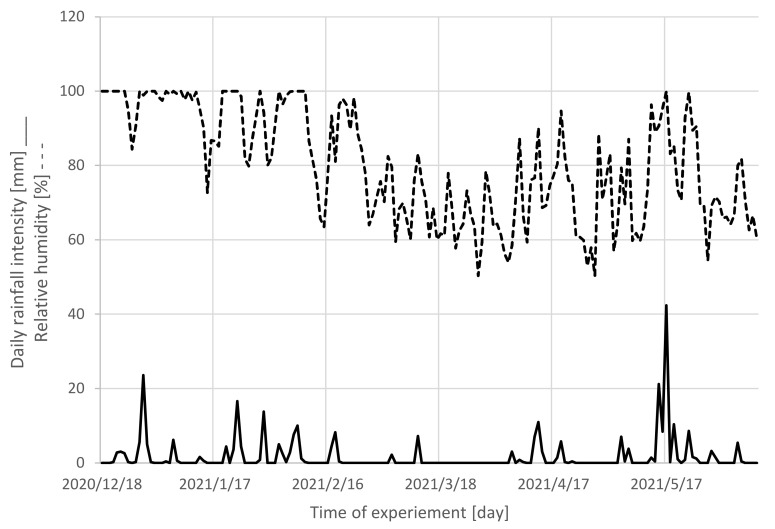
Daily rainfall intensity and relative air humidity during the 6-month-long storage.

**Figure 4:**
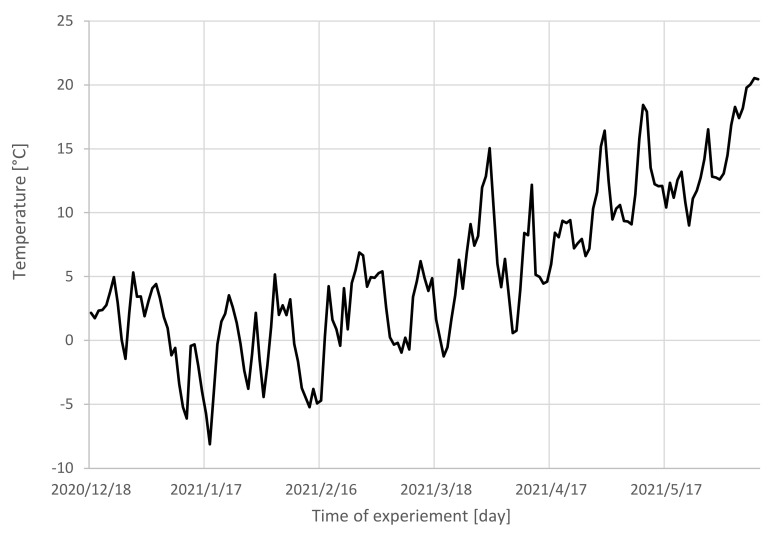
Air temperature in the roundwood environment during the 6-month-long storage.

The temperature of the roundwood environment during the storage, precipitation, as well as relative air humidity were recorded, and they correspond with the mass loss of wood (Figure 3 and Figure 4).

**Figure 5:**
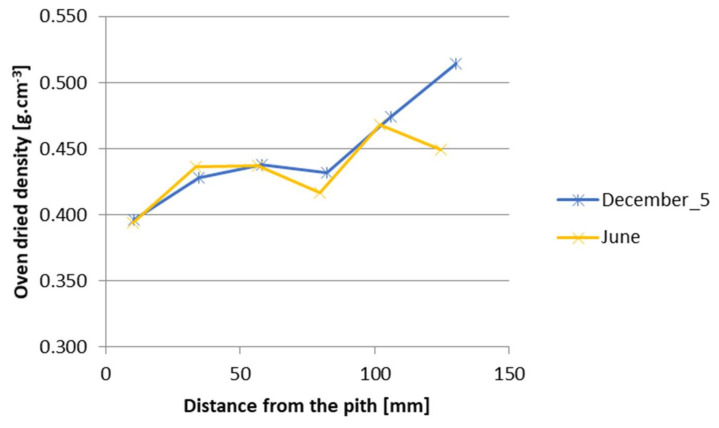
The change in the density in a dry state of parallel figures after the 6-month-long storage.

A decrease in density in a dry state after the 6-month-long storage is evident (Figure 5). The decrease in density can be observed on the surface of the roundwood, while in the centre—in the ripewood—only a slight change occurred (Figure 5).

After completing the Equations (4) and (5) with the measured data in a dry state and the position of the parallel figures, the value of DML of the spruce roundwood after the 6-month-long storage was 4.5%. Only a small amount of 0.37% was the loss of the mass of cytoplasm of living cells in the sapwood of the freshly logged spruce wood [9]. A gradual decrease in the dry substance was already visible after the first months of the storage (Table 1).

Gradual decline in factors affecting the *DML_j_* is also shown in Table 1.

**Figure 6:**
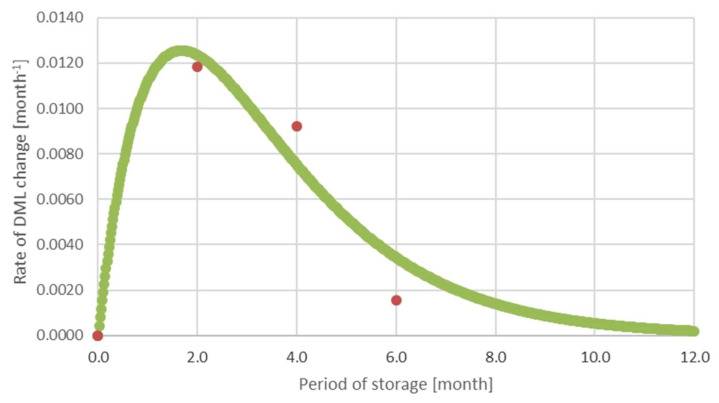
Estimation of the rate of DML change during the storage (red points denote the measured values, green line shows the graph of the Equation (6)).

A decrease in the rate of change of *DML_j_* indicates a gradual decline in a factor during storage after time in maximum. Firstly, there is an increase in the rate of change of *DML* during 6-month-long storage, then the value reaches the maximum and finally decreases (Figure 6). An estimation of the rate of the change in *DML* in time is approximated with the equation:

(6) $$\frac{dDML}{dt}=Ate^{-Bt}$$

where *t* is time, *A* and *B* are parameters, *e* is the base of a natural algorithm. The graph in Figure 6 shows inhibiting activity of the acting factors on *DML*.

The parameters *A* and *B* were calculated following the data mentioned in Table 1 about the change in *DML* for 2 months using the least-squares method. The index of correlation was 0.930.

**Figure 7:**
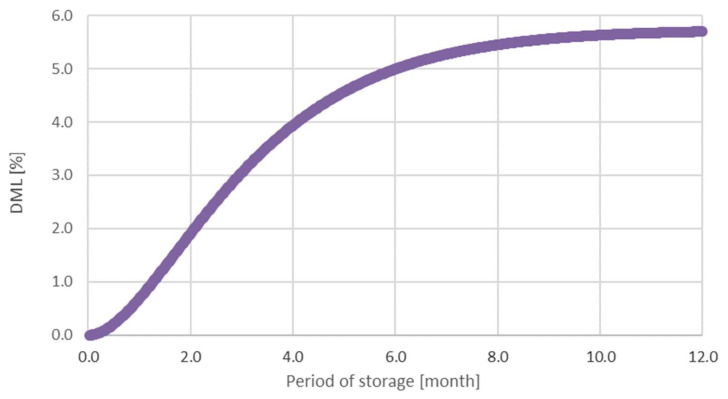
Dependence of DML on the time of the spruce wood storage.

*DML* is an integral of the rate of *DML* change in time (Figure 7) converted in time. The graph in Figure 7 shows the inhibiting activity of the factors on *DML*. The effect of the factors on *DML* mitigate after long-term activity.

After two months in storage, *DML* is estimated as 5.7%, which is a dry mass loss for one year. Similar results of 5.3% were observed by Tripathi et al. (2011) [29] after 12 months’ storage of eucalyptus wood.

The use of parallel figures when defining *DML* is considered a disadvantage. The change in the properties of spruce wood can result from the location in the trunk. The definition of *DML* according to the property not changing due to various factors seems to be an advantage. The more appropriate term seems to be “equilibrium moisture content”, defining the moisture state with the possibility of measuring the mass of a substance. However, the definition of moisture content as a wood property not dependent on how to achieve the state (sorption, absorption) is discussed. Therefore, the use of parallel figures seems to be the only solution to define *DML*.

**Figure 8:**
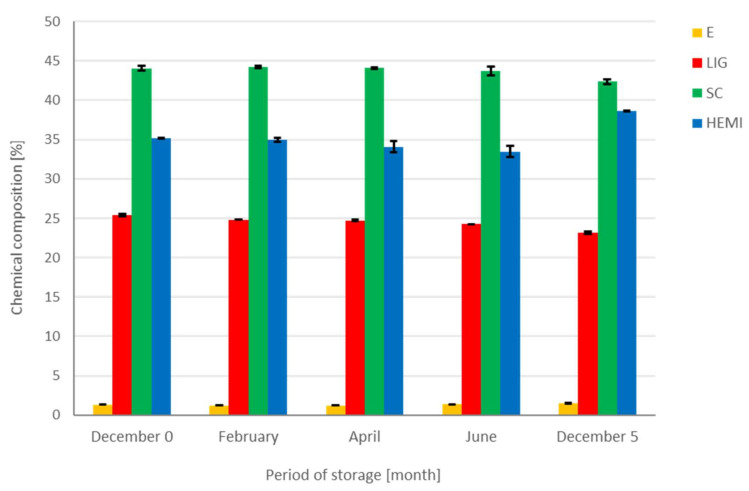
The change in the chemical composition of spruce wood during the storage (the whiskers at the tops of the bars mean standard deviations from arithmetic averages, the bars’ height).

Wood mass loss is a total characteristic. However, the chemical compounds in wood play an important role in processing data. Freshly felled spruce wood contained 1.51% of extractives (E), 80.97% holocellulose, 42.35% of cellulose (SC), 23.17% of lignin (LIG), and 38.62% of hemicelluloses (HEMI). The composition of spruce wood is mentioned in the work of Jeníček et al. [30], Malaťáková et al. [31], etc. The chemical composition of spruce wood after 2, 4, and 6 months of storage is shown in Figure 8. The percentage of hemicelluloses, cellulose, and lignin decreased during storage of spruce wood. Their decreases were probably caused by degradation. The decrease of cellulose and hemicelluloses percentage were observed by Tripathi et al. (2011) [29], also. They explained the change over time due to the free radical formation by UV, atmospheric oxygen, and degradation by bacteria, fungi etc.

**Figure 9:**
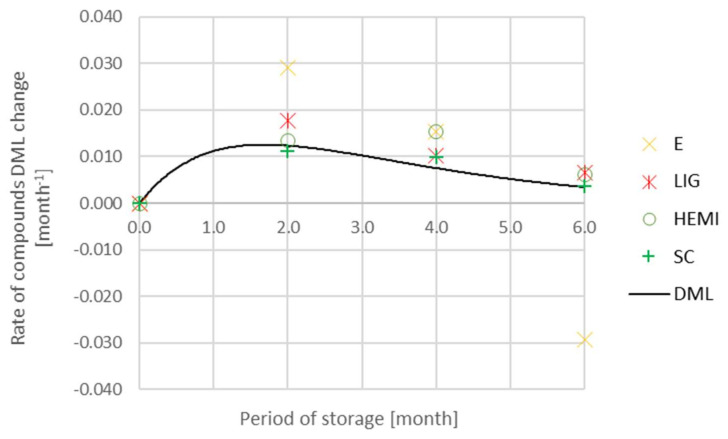
DML and the loss of chemical substances during storage.

Following the gathered results of chemical composition, it can be stated that the changes in the number of substances occur during the storage of spruce wood. The definition of DML is based on the density in the dry state, the Equations (4) and (5). However, the loss of chemical substances can be calculated when Equations (4) and (5) are completed with the mass concentration of chemical substances instead of the density in the dry state. The result of the calculation is shown in Figure 9.

In Figure 9, positive values show the loss, and the negative ones indicate an increase in the rate of the isolated substances from spruce wood compared to the sample isolated in December (December 0). After two months of storage, a major loss was observed in the case of extractives. On the contrary, the rate change of cellulose mass was the slowest (Figure 9). An increase in the extractives after 6-month-long storage was considered an exception. Thus, the loss of all isolated wood substances besides extractives is evident after six months of storage. However, the negative value of DML for extractives occurred also in the case of the samples December 0 and December 5 (Figure 1). The DML of the cellulose was similar to the DML of spruce wood. A decrease in DML of lignin and hemicelluloses was faster than in the case of spruce wood. The amount of hemicellulose and lignin decreased with dependence on the storage time, and it was higher than the value of DML of spruce wood during six months of storage. Hemicelluloses were the most unstable wood component and their degradation rate increased during four months of storage. During the wood storage, the extractives degraded, which resulted in changes in percentages, especially at the beginning of storage. A decrease in extractives the storage was observed by the authors Ramnath et al. [32], Günther et al. [33], and Giesel et al. [34] as well.

### Fourier-Transform Infrared Spectroscopy Analysis

**Figure 10:**
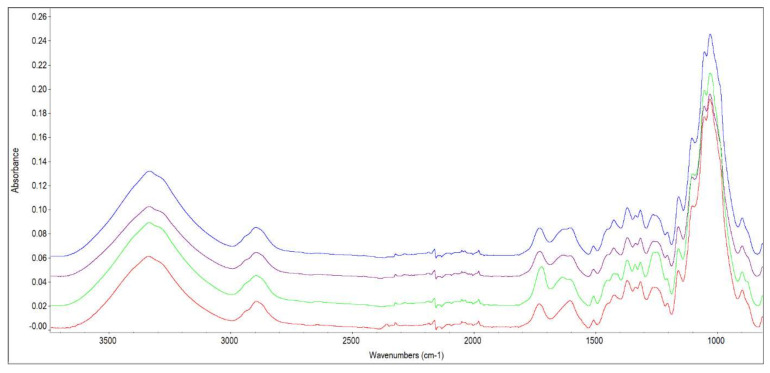
FTIR spectrum of holocellulose isolated from wood before and after 2, 4 and 6 months of storage (Note: blue line-December 0, magenta line-February, green line-April, red line-June).

**Figure 11:**
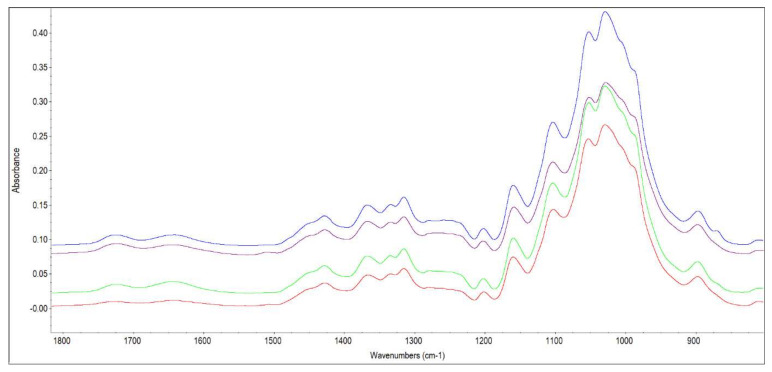
FTIR spectrum of cellulose isolated from wood before and after its 2-, 4- and 6- month-long storage (Note: blue line-December 0, magenta line-February, green line-April, red line-June).

ATR-FTIR absorption spectra were used to elucidate the changes in holocellulose (polysaccharides) (Figure 10) and cellulose (Figure 11) taking place in spruce wood before and after 2, 4, and 6 months of storage. The text describes only more significant changes in FTIR spectra of holocellulose (Figure 10). Holocellulose (cellulose + hemicellulose + residual lignin), which was isolated from the original and stored spruce wood (after 2, 4, and 6 months), showed absorbance changes ranging between 3200–3500 cm^−1^ (intra-molecular hydrogen-bonded cellulose region) and minimum band changes at 2800–2900 cm^−1^ (region of C-H vibrations in CH_2_ groups).

In the FTIR spectra with the peak at 1732 cm^−1^ (assigned to unconjugated carbonyl groups), an increase in absorbance, only between the original spruce wood before and after four months of storage, was due to an increase in carbonyl or carboxyl groups. Li et al. [35] studied the heat degradation of lignin in hardwood and softwood. An increase at 1720 cm^−1^ resulting from an increasing temperature was observed and it was concluded that it was due to the production of C=O bonds in lignin.

The band with the wavelength of 1635 cm^−1^ (conjugated C−O in quinones coupled with C=O stretching in various groups) almost disappeared after 6-month-long storage. It may happen because of the cleavage of aryl–ether bonds [36]. In the isolated holocellulose, an insignificant amount of lignin can be found that was also confirmed with the band at 1600 (belonging to aromatic skeletal vibration in lignin, and –C=O stretching, [37]) and 1508 cm^−1^ (C=C stretching of the aromatic skeletal vibrations in lignin, [38]). The intensity of the band increased at 1500 cm^−1^ only after 4-month-long storage. Some authors reported an increase in absorbance at this band in thermally treated wood [38,39]. The following increase in the band at the wavelength of 1241 cm^−1^ occurred in our research again after 4-month-long storage. Tjeerdsma and Militz [40] studied the FTIR spectra of holocellulose and lignin of heat-treated Fagus sylvatica and Pinus sylvestris. They concluded that an increase in the 1740 cm^−1^ band was only due to the lignin as there was no increase in holocellulose. These authors considered this increase to be due to the occurrence of esterification when the existent acid reacts with the hydroxyl groups of the cell wall material. If esterification occurs, there can be an increase in the band at 1240 cm^−1^ due to antisymmetric stretching vibration of the acetyl ester groups which also happened in our samples. The intensity in the case of the band at 1030 cm^−1^ (C−O−C stretching of a primary alcohol in polysaccharides) and 897 cm^−1^ (in-plane symmetric vibration of C-H) slightly decreased after wood storage may be a result of polysaccharide degradation.

The change in crystallinity of cellulose was observed in the cellulose isolated from wood using the FTIR method. The most interesting spectrum parts of cellulose are from 1420–1430 cm^−1^ (associated with the amount of crystalline structure of the cellulose) and in the region of 900–890 cm^−1^ (assigned to the amorphous region) [41], and in the region of 1365–1375 cm^−1^ (CH_2_ bending in cellulose and hemicelluloses). Furthermore, it is advised to be familiar with the ratio between the peaks assigned to these two cellulose states, namely 1430–1420/897 cm^−1^, which may indicate some information about the cellulose crystallinity degree, and it is referred to as the lateral order index (LOI) [42]. Moreover, the ratio between the peaks at ~1370 cm^−1^ and ~2900 cm^−1^ is regarded as the total crystalline index (TCI) [42]. Changes in LOI and TCI values are shown in Table 2.

The value of TCI and LOI in cellulose decreased due to the storage of felled wood. The intensity of bands used to calculate the value of TCI decreased by 37% at a wavelength of 1370 cm^−1^ and by 27% at the wavelength of 2900 cm^−1^, which resulted in a decrease in the value of TCI. This may indicate the loosening of the cellulose structure-crystallinity decreases [43]. The value of LOI decreased due to a decrease in the band intensity by 27% at the wavelength of 1423 cm^−1^ and a decrease in the band intensity by 17% at the wavelength of 894 cm^−1^. A decrease in values of TCI and LOI was determined in the work of [44].

## 4. Conclusions

In the study, the mass changes and changes in the chemical composition of wood after 2, 4, and 6 months of storage were measured. The dry wood substance loss after one year of storage was estimated using a regression model. The index of correlation was 0.930. The FTIR method was used to observe the change in cellulose crystallinity and structural changes in holocellulose. The estimated dry-mass loss of round spruce wood, harvested in the winter, in the one-year storage period was 5.7%. The use of parallel figures with similar properties is required to define DML. The content of hemicellulose and lignin is changed when there is a change in the density in the dry state. The rate of change of the DML of hemicellulose and lignin was always more considerable than the DML of spruce wood. A decrease in the DML of extractives after six months of storage was due to the change in the number of extractives after the height of a trunk. After two months of storage, the most significant decrease was observed in the case of extractives in comparison to isolated substances. The DML of cellulose was similar to the DML of spruce wood. Using the FTIR analysis we found that the total crystalline index (TCI) and lateral order index (LOI) in cellulose decreased because of the wood storage.

## Figures and Tables

**Table 1:** *DML_j_* after 2, 4 and 6 months of storage.

Time of Storage [Month]	0	2.0	4.0	6.0
*DML_j_*	0	0.0237	0.0185	0.00313

**Table 2:** Total Crystallinity Index and Lateral Order Index of cellulose after harvesting in December and 2, 4, 6 months of storage.

	TCI 1370 cm^−1^/2900 cm^−1^	LOI 1423 cm^−1^/894 cm^−1^
Harvesting in December	0.625	1.000
2 months of storage	0.619	0.995
4 months of storage	0.622	0.996
6 months of storage	0.560	0.880

## Data Availability

Data are available along with quotation of this article.
